# Benign multicystic peritoneal mesothelioma mimicking recurrence of an ovarian borderline tumor: a case report

**DOI:** 10.1186/1752-1947-6-126

**Published:** 2012-05-14

**Authors:** Shuji Takemoto, Ryosuke Kawano, Kazumi Honda, Aki Nakazono, Kazuhide Shimamatsu

**Affiliations:** 1 Department of Obstetrics and Gynecology, Omuta City Hospital, Takarasaka-machi 2-19-1, Omuta, Fukuoka, 836-8567, Japan; 2 Department of Pathology, Omuta City Hospital, Takarasaka-machi 2-19-1, Omuta, Fukuoka, 836-8567, Japan; 3 Department of Obstetrics and Gynecology, Kurume University School of Medicine, Kurume, Fukuoka, 830-0022, Japan

## Abstract

**Introduction:**

Benign multicystic peritoneal mesothelioma is an extremely rare tumor that occurs mainly in women in their reproductive age. Its preoperative diagnosis and adequate treatment are quite difficult to attain.

**Case presentation:**

Our patient was a 23-year-old Japanese woman who had a history of right oophorectomy and left ovarian cystectomy for an ovarian tumor at 20 years of age. The left ovarian tumor had been diagnosed on histology as a mucinous borderline tumor. Two years and nine months after the initial operation, multiple cysts were found in our patient. A laparotomy was performed and her uterus, left ovary, omentum and pelvic lymph nodes were removed due to suspicion of recurrence of the borderline tumor. A histological examination, however, revealed that the cysts were not a recurrence of the borderline tumor but rather benign multicystic peritoneal mesothelioma. There were no residual lesions and our patient was followed up with ultrasonography. She remains free from recurrence nine months after treatment.

**Conclusion:**

We report a case of benign multicystic peritoneal mesothelioma mimicking recurrence of an ovarian borderline tumor. Benign multicystic peritoneal mesothelioma should be suspected when a multicystic lesion is present in the pelvis as in the case presented here, especially in patients with previous abdominal surgery.

## Introduction

Benign multicystic peritoneal mesothelioma (BMPM) is a rare peritoneal condition which occurs most frequently in women with a history of abdominal surgery. It is characterized by a slowly progressive process and a high rate of relapse after surgical resection [1]. Preoperative or intraoperative diagnosis has been shown to be difficult, especially the differential diagnosis of an ovarian tumor. It is also difficult to manage this disease appropriately because of its rarity.

## Case presentation

A 20-year-old Japanese woman presented to a local hospital with lower abdominal pain. She had mental retardation and epilepsy, and was unable to indicate her intentions. She was found to have a multicystic tumor in her pelvic cavity and was referred to our gynecologic department. Her lower abdomen was soft and showed slight swelling without tenderness. Magnetic resonance imaging revealed that a multicystic tumor occupied her abdominal cavity. Because the tumor did not have a solid component or enhancement with gadolinium enhancement and tumor markers were normal (carbohydrate antigens [CA] 125, CA19-9 and carcinoembryonic antigen), a benign ovarian tumor was suspected. A laparotomy was performed, unexpectedly revealing swelling of her bilateral ovaries. The right ovarian tumor was the size of an adult head and the left ovarian tumor was the size of a fist. A right oophorectomy and left ovarian cystectomy were performed. Gross findings were that the bilateral ovarian tumors seemed benign without a solid or papillary component. The pathological diagnosis of the right ovarian tumor was mucinous cystadenoma; however, the pathological diagnosis of the left ovarian tumor was an intestinal type mucinous borderline tumor. Our patient did not receive any adjuvant therapy and we planned to follow up every three months.

**Figure 1:**
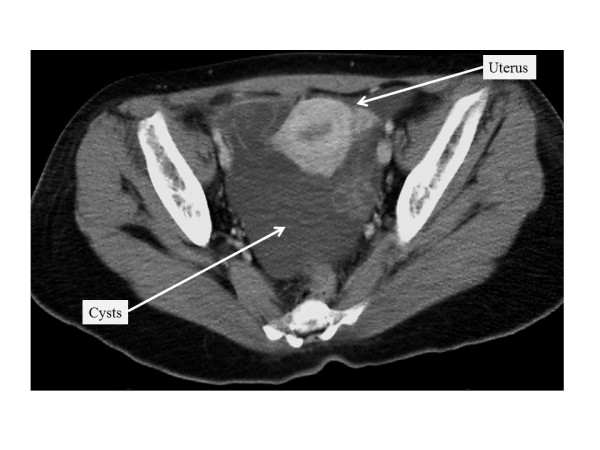
**Computed tomography of the pelvis two years and nine months after the first surgery.** There were multiple cysts in the pelvis.

**Figure 2:**
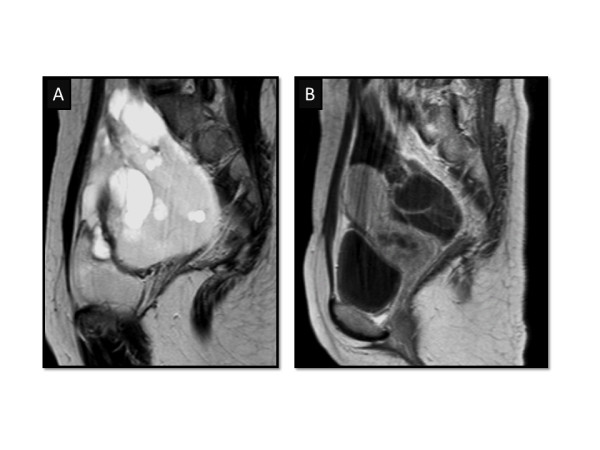
**Magnetic resonance imaging of the pelvis on a (A) T2-weighted and (B) gadolinium enhanced T1-weighted image.** The cyst walls were partly enhanced with gadolinium administration.

Two years and nine months after the primary surgery, a multilocular tumor, approximately 10 cm in diameter, was detected in her pelvic cavity on follow-up ultrasonography. A computed tomography scan revealed a multicystic tumor in her pelvic cavity, without contrast by a radiopaque agent (Figure 1). Magnetic resonance imaging revealed round cystic lesions in her pelvis, with low to intermediate intensity on T1-weighted images and high intensity on T2-weighted images (Figure 2). A small part of the cyst wall was slightly enhanced with the administration of gadolinium. The preserved left ovary was not separately identified. Therefore, a recurrent ovarian borderline tumor was suspected.

Our patient could not give her own opinion regarding her disease so her parents made all decisions regarding her treatment. Our patient had suffered from dysmenorrhea for a long time and her parents therefore requested removal of her uterus and remaining ovary.

**Figure 3:**
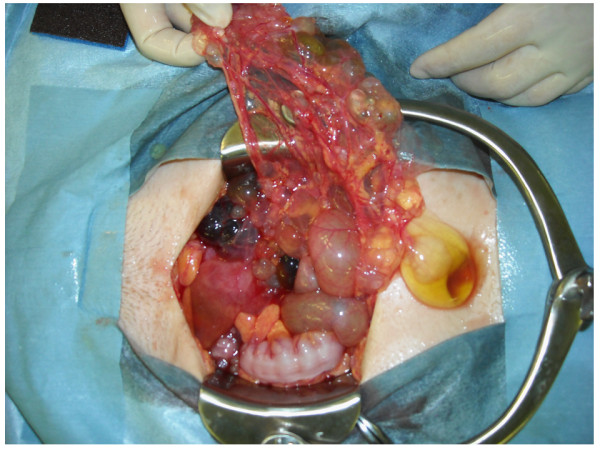
**Intraoperative finding of the pelvis.** There were multiple cysts adhering to the surfaces of the ovary, mesentery, mesocolon and pelvic wall.

Our patient then underwent a second surgery and a multicystic tumor measuring 10 × 10 cm was found in her pelvic cavity. The tumor consisted of many non-isolated cysts of diverse sizes (Figure 3), which adhered to the surface of her ovary, mesentery, mesocolon and pelvic wall. Slight transparent ascites was present. Her uterus was normal and her left ovary was thumb-sized without swelling. A hysterectomy, left oophorectomy, omentectomy, pelvic lymph node biopsy, appendectomy and tumorectomy of her abdomen were performed. Complete resection of the lesion was achieved.

**Figure 4:**
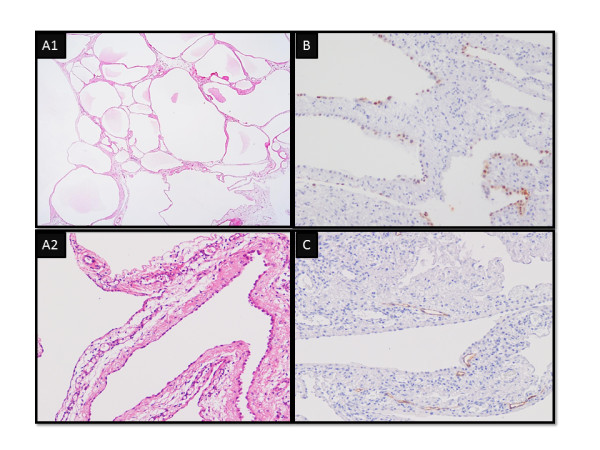
**Histological findings of the cysts. (A1, A2)** Hematoxylin and eosin staining; **(B)** calretinin staining; **(C)** D2-40 staining. The lining cells of the cyst wall were positive for calretinin and negative for D2-40.

On histology, epithelium with a mucinous component was not seen in the cyst or cyst wall (Figure 4). In the removed left ovary, a tiny component of mucinous cystadenoma was present though neither a mucinous borderline tumor nor a mucinous adenocarcinoma was present. Immunohistochemical staining of the lining cells of the cyst wall were positive for calretinin and Wilms’ tumor protein and negative for D2-40. The removed uterus, lymph nodes and omentum were normal. Our patient was finally diagnosed with BMPM. She recovered uneventfully and was discharged from the hospital on the seventh day after the operation. Our patient received estrogen replacement therapy and remains free from recurrence nine months after the second operation.

## Discussion

Peritoneal mesothelioma is an uncommon lesion that originates from mesothelial cells lining the human body cavities. The incidence is approximately one per 1,000,000 and makes up approximately one-fifth to one-third of all mesotheliomas [2]. Of these peritoneal mesotheliomas, BMPM was reported to constitute about 3% to 5% and only about 130 cases of BMPM have been reported [3], beginning with the first report by Mennemeyer and Smith in 1979 [4]. The pathogenesis of BMPM is controversial. Some authors believe that the lesion is neoplastic because two cases were reported as malignant transformations of BMPM [3,5]. Others believe the pathogenesis to be a reactive process because of the close relationship between BMPM and a history of abdominal surgery, such as hysterectomy and tubal ligation. In addition, endometriosis may result from a peritoneal reaction to chronic stimuli [1,6,7]. Unlike pleural mesothelioma, BMPM is not associated with prior exposure to asbestos.

Among women who wish to have their fertility preserved, it is quite worthwhile to distinguish BMPM from an ovarian tumor intraoperatively. In a review of seven cases of mesothelial tumor, four cases with a benign mesothelial tumor were initially suspected to have ovarian tumors before surgery [8]. Although a few cases have been diagnosed as BMPM before surgery by aspiration cytology [9] or during surgery by histological diagnosis using a frozen section [10], it is difficult to correctly diagnose and treat this disease. Surgery is reported to be the only effective treatment for BMPM, and adjuvant chemotherapy and radiotherapy are not indicated because these tumors have a prevailing benign character [1]. Patients do, however, have a 30-50% risk of recurrence, from one month to many years postoperatively [10]. Therefore, it is necessary to follow up after the resection of BMPM.

The influence of estrogen on BMPM is not well understood. The reduction of cyst volume and cyst growth after therapy with gonadotropin-releasing hormone agonist [11] and the anti-estrogen agent tamoxifen [12] lend further support to the theory that BMPM is an estrogen-dependent condition. The biological rationale for this response, however, remains unexplained; immunohistochemical detection of estrogen receptors and progesterone receptors was reported to be uncommon [6]. Moreover, BMPM has been reported even in male patients [13]. As mentioned above, our patient is now receiving estrogen replacement therapy in order to prevent osteoporosis or symptoms occurring due to the lack of ovarian function.

## Conclusion

We present a case of BMPM mimicking the recurrence of an ovarian borderline tumor. BMPM should be suspected when a multicystic lesion is present in the pelvis as in the case presented here, especially in patients with previous abdominal surgery. It is necessary to closely monitor for recurrence after surgery for mucinous borderline tumors in particular, because they seem to be the most difficult to distinguish. More case series are needed in order to reveal this unknown etiology.

## Consent

The patient had mental retardation and epilepsy, and was unable to indicate her intentions. Therefore, written informed consent was obtained from the patient’s parents for publication of this manuscript and any accompanying images. A copy of the written consent is available for review by the Editor-in-Chief of this journal.

## Competing interests

The authors declare that they have no competing interests.

## Authors’ contributions

ST, KH and RK performed the operation, and they and AN performed the postoperative management. KS performed the histological examination of the patient’s disease. ST was the patient’s attending doctor and was a major contributor in writing the manuscript. All authors read and approved the final manuscript.
